# Inflammation-associated premetastatic niche formation

**DOI:** 10.1186/s41232-022-00208-8

**Published:** 2022-07-03

**Authors:** Atsuko Deguchi, Yoshiro Maru

**Affiliations:** Department of Pharmacology, Tokyo Women’s Medical University, 8-1 Kawada-cho, Shinjuku-ku, Tokyo, 162-8666 Japan

**Keywords:** Cancer, Metastasis, TLR4, Premetastatic niche, S100A8, Inflammation, Tumor microenvironment

## Abstract

Metastasis remains the leading cause of cancer-related death. In 1889, Stephen Paget originally proposed the theory “seed-and-soil.” Both cancer cell-intrinsic properties (“seed”) and fertile microenvironment (“soil”) are essential for metastasis formation. To date, accumulating evidences supported the theory using mouse models. The formation of a premetastatic niche has been widely accepted as an accel for metastasis. Similar to tumor microenvironment, various types of cells, such as immune cells, endothelial cells, and fibroblasts are involved in premetastatic niche formation. We have discovered that primary tumors hijack Toll-like receptor 4 (TLR4) signaling to establish a premetastatic niche in the lung by utilizing the endogenous ligands. In this review, we discuss the mechanisms that underlie inflammation-associated premetastatic niche formation upon metastasis, focusing especially on myeloid cells and macrophages as the cells executing and mediating complicated processes.

## Background

Metastasis consists of a continuous and multi-step biological process, in which a small population of cancer cells with highly invasive and metastatic potential secede from their primary tumors, induce degradation of the extracellular matrix (ECM), intravasate into the blood or lymphatic vessels, and extravasate to and colonize to distant organs. The theory of “seed and soil” proposed by Paget [1] has been widely accepted. To date, secreted cytokines, such as vascular endothelial growth factor (VEGF), stromal cell-derived factor 1(SDF-1), transforming growth factor b (TGF-b), and tumor necrosis factor (TNF), which form premetastatic niches, induce mobilization of bone marrow-derived cells. The recruitment of vascular endothelial growth factor receptor 1 (VEGFR1)^+^ hematopoietic bone marrow progenitors, CD11b^+^ myeloid cells and suppressive immune cells, initiates the pre-metastatic niches and thereby promotes metastasis [2]. We previously identified S100A8 which was upregulated in the lungs of tumor-bearing mice [3]. S100A8 is one of the known endogenous ligands of TLR4 [4].

Toll-like receptors (TLRs), the best-characterized family of pattern recognition receptors, were originally characterized for their ability to respond to exogenous pathogen-associated molecular patterns (PAMPs) that include bacterial lipopolysaccharide (LPS), bacterial diacylated and triacylated lipopeptides, bacterial flagellin, bacterial and viral unmethylated CpG-containing DNA motifs, and viral single- and double-stranded RNA [5]. Among TLRs, TLR4 activation via LPS is essential for the host defense against gram-negative bacteria. In addition to PAMPs, TLR4 also recognizes danger-associated molecular patterns (DAMPs). Heat-shock protein (Hsp) 60 [6], Hsp70 [7], high-mobility group box 1 (HMGB1) [8], S100A8/S100A9 [4], serum amyloid A3 (SAA3) [9, 10], Fetuin-A [11], defensin β [12], fibrinogen [13], and fibronectin [14], as well as polysaccharides such as hyaluronan [15], heparan sulfate [16], biglycan [17], and decorin [18], appear to be endogenous ligands of TLR4. The ligand-induced dimerization of TLRs triggers the recruitment of adaptor proteins to intracellular TIR (Toll/interleukin-1 receptor) domains to initiate signaling. Signaling cascades via the TIR domains are mediated by specific adaptor molecules, including MyD88, MAL (also known as TIRAP), TRIF, and TRAM. TLR4 associates with co-receptor MD-2 and LPS binding to MD-2 induces the dimerization of TLR4 [19]. Intriguingly, accumulating evidence suggest that DAMP-mediated signals can promote metastasis. Initially, we thought that S100A8 would mainly play a role in premetastatic niche formation, but it has been reported that various types of cancer cells also by themselves express TLR4 at the cell surface. To date, S100A8 expression, or overexpression of TLR4, was observed in various cancers.

## Myeloid derived suppressor cells (MDSCs) in metastasis

MDSCs are immature myeloid cells derived from bone marrow, recruit to primary tumor sites and distant organs, which contribute to various immune responses. The recruitment of MDSCs is a crucial step for premetastatic niche formation [20–22]. The ability of immunosuppression is one of the major characteristics of MDSCs. In mice, MDSCs can be divided at least into two populations, namely granulocytic/polymorphonuclear MDSCs (PMN-MDSCs) and monocytic MDSCs (M-MDSCs). In addition, early MDSCs (eMDSC) have been identified in humans. Classical myeloid cell activation is mainly driven via TLR activation. Both M-MDSCs and PMN-MDSCs express S100A8/S100A9. PMN-MDSCs in the premetastatic niches may contribute to the escape of tumor cells by suppressing immune cells, inducing matrix remodeling, and promoting angiogenesis, which in turn promote colonization of tumor cells. In mice, M-MDSCs are defined as CD11b^+^Ly6G^–^Ly6C^high^, and PMN-MDSCs are defined as CD11b^+^Ly6G^+^Ly6C^low^. It should be noted that these markers are only applicable for mice, but not for humans. In humans, M-MDSCs are originally defined as either CD14^+^HLA^−^DL^low^ or CD11b^+^CD14^−^CD33^+^CD15^−^ cell populations, whereas PMN-MDSCs are defined as CD11b^+^CD14^−^CD15^+^ or CD11b^+^CD14^−^CD66^+^ cells.

## Tumor associated macrophages (TAMs) and metastasis-associated macrophages (MAMs)

TAMs, one of the most abundant inflammatory stromal cells in the tumor microenvironment, exhibit predominantly M2-like pro-tumor phenotype rather than M1-like anti-tumor phenotype, and their importance in metastasis is well established [23]. The subpopulation of recruited MDSC from the bone marrow differentiates to TAMs. Similar to MDSC, TAMs can induce immunosuppressive cytokines. Blockade of the CSF1–CSF1R with anti-CSF1 antibodies or a CSF-1R inhibitor with chemotherapy enhanced the therapeutic efficacy, inhibited metastases, and increased the recruitment of CD8 T cells in tumors [24].

Moreover, metastasis-associated macrophages (MAMs) have been identified in murine experimental models as F4/80^+^ CD11b^+^CD11c^−^Ly6C^−^ cells [25]. Of note, both TAMs and MAMs are originated from the bone marrow, but not residential macrophages. Lu et al. reported that TAM interacted with cancer stem cells, which would maintain cancer stemness [26]. These results indicate that the interaction in primary tumor sites contributes to maintain an invasive phenotype.

## TLRs in premetastatic niche formation

**Fig. 1:**
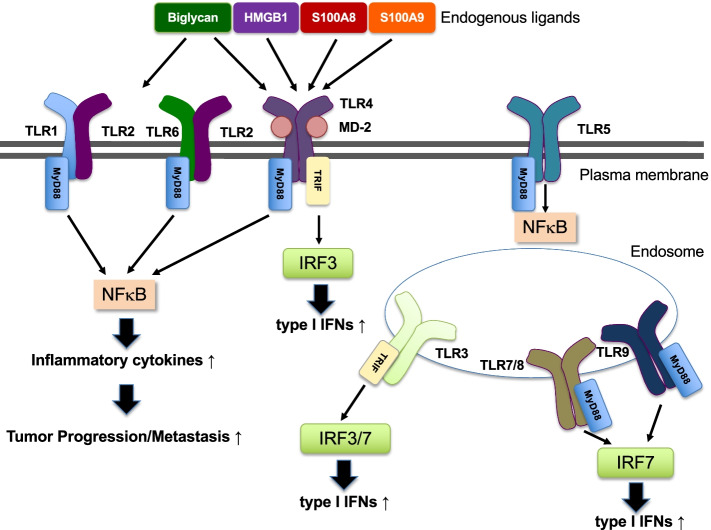
TLRs involved in metastasis. TLR2 associates with TLR1 or TLR6. TLR4 recognizes LPS or endogenous ligands (i.e., biglycan, HMGB1, S100A8, S100A9). TLR3 is implicated in the recognition of viral dsRNA or exosomal RNA. TLR2, TLR3, or TLR4 are involved in cancer metastasis

TLR2, TLR3, and TLR4 have been reported that TLR-mediated signaling involves premetastatic formation (Fig. 1). We focused on these three receptors and their ligands to discuss the mechanisms by referring to their findings.

### TLR2

Kim et al. firstly reported that versican induces metastasis in a TLR2-dependent manner. Versican is one of the TLR2/6 endogenous ligands, which can activate TLR2 downstream signaling. The deficiency of TLR2 in host cells significantly reduced pulmonary metastasis [27]. Moreover, Gao et al. found that bone marrow-derived CD11b^+^Gr1^+^ myeloid progenitor cells were recruited to premetastatic lung niches in the PyMT spontaneous breast cancer model [28]. In these niches, a specific subpopulation of myeloid cells, CD11b^+^Ly6C^high^ produced the ECM proteoglycan versican that stimulated mesenchymal-to-epithelial transition of metastatic tumor cells, which induced proliferation and hence the development of macrometastases [28].

### TLR3

The secreted molecules by the primary tumor also contain exosomal RNAs. Liu et al. reported that TLR3 in alveolar type II (AT-II) epithelial lung cells by tumoral-exosome-derived non-coding snRNA-mediated TLR3 activation in alveolar type II epithelial lung cells contributes to neutrophil (possibly contains PMN-MDSCs) infiltration and lung metastasis [29]. TLR3-deficient mice show reduced pulmonary metastasis in the spontaneous metastatic mouse models. These results suggest that not only immune cells but also other host cells, in this case resident alveolar epithelial cells, can involve in premetastatic niche formation.

### TLR4

**Fig. 2:**
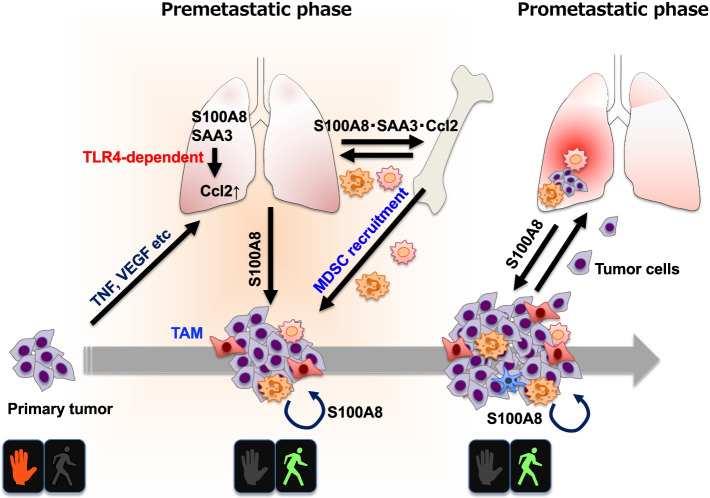
The recruitment of myeloid-derived cells is a crucial step in metastasis. In the early phase of the primary tumor, it is not ready for myeloid-derived cells (inflammatory monocytes, MDSCs) to recruit primary tumor sites and distant organs (in this case, the lungs; do not walk). Once tumors develop to a certain size and acquire invasiveness, the primary tumor microenvironment secretes cytokines/chemokines (i.e., VEGF, TNFα, and TGF-β) to produce S100A8 in the lungs. These chemokines, such as Ccl2, S100A8/S100A9, can send a signal to myeloid-derived cells in bone marrow as “walk”

We demonstrated that secretion of VEGF-A, TNFα, and TGF-β by primary tumors induces expression of S100A8/S100A9 in the lung, which causes a fertile microenvironment for metastasis (Fig. 2). S100A8 is known to be an endogenous ligand of TLR4 which can induce serum amyloid A (SAA) 3 in the premetastatic lung. Essentially, among all TLR series, only TLR4 can activate two downstream signalings upon activation by PAMPs. This resulted in the accumulation of CD11b^+^ myeloid cells and propagated a positive feedback loop for further chemoattractant secretion, all of which led to enhanced metastasis in the lung [3, 9]. In addition to the induction of S100A8 in the premetastatic lung, upregulation of S100A8 in the tumor microenvironment, which involves into the recruitment of myeloid cells in lungs from the bone marrow to primary tumors. Moreover, we further found that TLR4 knockdown cells grew slower than parental cells in vivo, whereas TLR4 knockdown cells grew similar to parental, or scramble shRNA-infected cells in 2D culture, indicating that tumor microenvironmental factors secreted by host immune cells are necessary to induce TLR4-mediated tumor growth [30]. To investigate whether TLR4 inhibition is sufficient to suppress tumor progression, Eritoran was used for further study. Eritoran is a structural analog of the lipid A from *R sphaeroides* (RsLA), competitively binds to TLR4/MD-2, which results in inhibition of LPS-induced inflammatory responses [31]. We found that Eritoran blocked S100A8-mediated TLR4 activation and reduced TAMs and CD11b^+^Ly6C^++^Ly6G^−^ populations in tumor microenvironment. Conversely, the treatment with Eritoran in tumor-bearing mice significantly increased intratumor infiltrating CD8^+^ T cells compared with the tumor-bearing control mice. The suppression of T cell function by MDSCs more likely depends on Arginase-1 expression [30].

Fukata et al. reported that mice with constitutive active TLR4 in epithelial cells were highly prone to colitis and colitis-associated colorectal tumor [32]. Stimulation of TLR4 signaling induces cyclooxygenase-2, which causes PGE_2_ production followed by upregulation of amphiregulin. In skin cancer, TLR4 also appears in keratinocytes and levels up during their progression to squamous cell carcinoma (SCC), whereas silencing of TLR4 led to the blockade of UV stress [33]. Moreover, Fleming et al. recently reported that tumor cell-derived extracellular vesicles convert normal myeloid cells into functional MDSCs by regulating the expression of PD-L1 via TLR4 signaling [34]. TLR4-induced TGF-β expression has also been associated with the transformation of fibroblast into cancer-associated fibroblast (CAF) in the tumor microenvironment, facilitating cancer cell proliferation and tumor growth [35, 36]. Therefore, targeting the premetastatic niche-promoting molecular components to prevent metastasis may be an attractive approach for cancer therapeutics.

#### S100A8/S100A9

S100A8 belong to the S100s family, exclusively expressed in vertebrates and characterized by two calcium-binding EF-hand motifs: a carboxyl-terminal EF-hand (high affinity to calcium) and an N-terminal EF-hand (low affinity to calcium) and connected by a central hinge. Calcium-binding within the EF-hand motif occurs in response to increased intracellular calcium concentrations and causes conformational changes that expose a wide hydrophobic cleft that interacts with target proteins. Like most S100 proteins, S100A8 can form as a heterodimer/heterotetramer with S100A9 and as a homodimer or monomer [37]. Vogl et al. originally reported that S100A8-mediated cytokine induction was mediated by TLR4 [4]. Moreover, we found that carboxyl terminal region of mouse S100A8 has a high affinity to TLR4/MD-2 and that S100A8 induced inflammatory cytokines/chemokines in a TLR4-dependent manner [30]. Although it has been reported that the S100A8/S100A9 heterodimer complex promotes colon tumor progression via the RAGE-dependent pathway [38], S100A8-mediated TLR4 activation in host cells could play a role in inflammatory cytokine/chemokine production.

#### Biglycan

Secreted proteoglycans such as biglycan, decorin, and versican can act as endogenous ligands of TLRs. Biglycan, a member of the family of small leucine-rich proteoglycan, is a ubiquitous ECM component. Biglycan acts as an endogenous ligand of both TLR2 and TLR4. Schaefer et al. reported that biglycan can bind to both TLR2 and TLR4 directly, but not TLR3, 5, 7, or 8 [17]. Recently, Maishi et al. reported that biglycan secreted from tumor endothelial cells induced tumor cell migration through NFκB and ERK2 activation via TLR2/4, which resulted in tumor metastasis [39]. In breast, lung, and colorectal cancers, high expression of biglycan correlated with poor prognosis [39]. In addition, Cong et al. found that biglycan-deficient mice showed vascular normalization in the tumor microenvironment, which enhances the efficacy of chemotherapy [40].

#### HMGB1

High-mobility group box 1 (HMGB1), as a member of the HMG family, predominantly locates in the nucleus as a DNA chaperone, which also translocates to cytoplasm, as well as the outside of the cell as a DAMP molecule. Because HMGB1 knockout mice die shortly after birth, HMGB1 is essential for development. HMGB1 is ubiquitous in mammals and generally abundant. HMGB1 is secreted from dead cells or activated immune cells, enterocytes, hepatocytes, and possibly several other cell types during infection, injury, or severe stress [41].

HMGB1 comprises two DNA-binding domains (so-called Box A and Box B) and acidic C-terminal tail. Since HMGB1 lacks a signal sequence, secretion occurs with the fusion of vesicles with the plasma membrane after an inflammatory signal. According to this, NLRP3 inflammasome might have an important role in the secretion of HMGB1 [42]. HMGB1 interacts not only with TLR4, but also with TLR2, and the receptor for the advanced glycation end product (RAGE). Yang et al. reported that HMGB1 failed to induce inflammatory cytokines in TLR4-deficient macrophages. It is still unclear how HMGB1 chooses a specific receptor, but it has been reported that TLR4 but not RAGE is required for cytokine release [8] and that RAGE is involved in cell migration [43].

Among endogenous TLR4 ligands, HMGB1 is studied in detail by using HMGB1 protein of endotoxin-free grade or synthetic peptide (B Box (89–108)). Endotoxin-free grade of HMGB1 or B Box peptide binds to TLR4/MD-2 in a concentration-dependent manner, whereas Cys106Ala peptide failed to bind to TLR4/MD-2. Therefore, Cys106 residue of HMGB1 is required for the binding to TLR4/MD-2 and activation of inflammatory cytokine production [8]. In addition to the importance of Cys106, TLR4 activation by HMGB1 requires a concomitant disulfide Cys23-Cys45 linkage [44]. Accordingly, the redox state is important for HMGB1-dependent TLR4 activation. More recently, Yang et al. developed tetramer FSSE to inhibit HMGB1-MD-2 interaction, while sparing PAMP, like LPS, signaling through TLR4 [45].

The blockage of HMGB1 can be applied as a therapeutic purpose against cancer disease? Nadatani et al. reported that administration of non-steroidal anti-inflammatory drugs induced small intestinal damage through a TLR4-dependent manner, and was associated with an increase of HMGB1 in both intestine and serum. The blockage of HMGB1 with neutralizing antibodies prevented both tissue damage and inflammatory cytokine production [46]. It has been reported that HMGB1 increased MDSC-mediated IL-10 production [47]. These findings suggest that a strategy of HMGB1 blockage is one of the useful clinical approaches against inflammatory-related diseases.

## S100A8-TLR4/MD-2 signaling can be a therapeutic target against cancer progression/metastasis?

As mentioned above, we found that Eritoran, a TLR4 inhibitor, inhibits tumor growth by immunomodulation and vascular normalization. Administration of anti-S100A8 neutralizing antibodies also suppresses tumor progression by the suppression of MDSC recruitments [30]. Of note, the neutralizing antibodies inhibit not only lung metastasis, but also liver metastasis (Deguchi, unpublished data). Initially, we thought that S100A8 mainly would play a role in premetastatic niche formation, but it has been reported that various types of cancer cells also by themselves express TLR4 at the cell surface.

Therefore, S100A8-TLR4/MD-2 may play crucial roles in both host cells and primary tumors. Accordingly, the S100A8-TLR4/MD-2 axis could be a novel therapeutic target against cancer progression and metastasis by immunomodulation and vascular normalization.

## Existence of metastasis-suppressive cell population in premetastatic phase

We have learned that premetastatic niche formation contributes to metastasis; however, it is likely that some suppressive effects would also exist to maintain homeostasis during the premetastatic phase. Moreover, until recently, the mechanisms of metastasis suppression are poorly understood. The bone marrow-derived Gr-1^+^ myeloid cells which express thrombospondin-1 suppress metastasis, and primary tumor-derived prosaposin play a key role in thrombospondin-1 induction in the Gr-1^+^ cells [48]. Of note, the expression of prosaposin in prostate cancer was positively correlated with increased overall survival. More recently, we investigated the suppressive role of metastasis during the premetastatic phase and identified the population of B220^+^CD11b^+^NK1.1^+^ cells as a pulmonary metastasis suppressor. These results suggested that hepato-entrained B220^+^CD11b^+^NK1.1^+^ cells suppressed lung metastasis [49]. These findings would provide a novel clue for therapeutic targets against metastatic tumors.

## Conclusions

We now realize that many types of immune cells complexly contribute to premetastatic niche formation for immune escape. Chemoresistance or recurrence are major causes of cancer-related death. Elucidation of the underlying mechanism(s) can contribute to establishing suitable therapeutic targets. Of note, metastatic tumors tend to disseminate to specific distant organs. For example, aggressive colon cancers typically metastasize to the liver. We found that S100A8 plays an important role in the premetastatic niche formation, but not only to the lungs specifically. Our results suggested that S100A8 universally induce the cell mobilization of MDSCs and other inflammatory monocytes from the bone marrow. As mentioned above, S100A8 is one of the major inflammatory cytokines in acute and chronic inflammation. Intriguingly, S100A8 expression is known to be upregulated in severe COVID-19 patients [50, 51]. Therefore, in addition to cancer disease, it is likely that S100A8/TLR4/MD-2 axis can be a promising therapeutic target against other inflammation-associated diseases.

## Data Availability

Not applicable.

## Abbreviations

TRIF: TIR domain-containing adaptor protein-inducing interferon β
TRAM: TRIF-related adaptor molecule
MyD88: Myeloid differentiation factor 88
MAL: MyD88 adaptor-like
CSF1: Colony-stimulating factor 1
CSF1R: Colony-stimulating factor 1 receptor
NLRP3: NLR family pyrin domain containing 3
PGE_2_: Prostaglandin E2
